# On the Ectrotic Treatment of Small-Pox by Tincture of Iodine

**Published:** 1846-08

**Authors:** 


*On the Ectrotic Treatment of Small-Pox by Tincture of Iodine. Being an extract of a letter from* Dr. Samuel Jackson, *of Philadelphia, (late of Northumberland,) to* Professor Dunglison.

In April, 1845, I was led to make an experiment of aborting small-pox by the tincture of iodine, from contemplating its wonderful influence over erysipelas. I applied it to one arm of a child eleven months old, in confluent small-pox, on the third day of the eruption, and to the arm which appeared the worst, rubbing it freely on with a sponge, three times that day and twice the next. On the 11th day, when the pocks over the whole body were at their height, elevated with hard bases, those of the medicated arm were entirely flat, with thin, purulent matter under the dead cuticle, without any swelling of the part. In this state was the disease when I showed the case to Drs. Bond and Nancrede, who agreed with me that there was a complete abortion. There are, however, some very slight pits now to be seen, but they are very inconsiderable when compared with those on the other arm.

I have not had an opportunity of repeating the experiment, for during the late epidemic I saw nothing but varioloid, and that so slight that no trial could be made. I mentioned the child's case to a number of physicians, but I do not know that any of them tried the medicine, except Drs. Goddard and Sargent, whose written reports I send you.

Dr. Sargent used the iodine on one side of the face in 25 cases —" the swelling, soreness, and tenderness were very much less than on the side not covered ; each pock remained flattened ; but I cannot say that it prevented pitting."

Dr. Goddard writes that he tried the medicine in five cases— " not one of the patients shows the least pit or mark ; none of them had been vaccinated, and the disease was confluent in most of them."

Dr. Sargent's experiments are not as favourable as Dr. Goddard's and my own—possibly from using a feebler medicine. That which I used was taken from my own closet, made by myself.

One advantage of this treatment is, that it removes the cuticle and leaves the part free from those disgusting discolorations which commonly remain for months.

It might be well to consider how far it would be prudent to extend the application over the body, in order to mitigate the disease, in malignant or even in severe cases. No fair trial can be had without applying it on the first day of the eruption and continuing it for several days, say 5 or 6.

I have found the same medicine an admirable remedy in the irritable ulcer with inflamed surface, and erysipelatoid margins. It soon kills the cuticle, and with this the whole inflammation disappears, when a little lunar caustic to the ulcer disposes it to granulate.

